# Integrated transcriptomic and proteomic analyses reveal the mechanism of easy acceptance of artificial pelleted diets during food habit domestication in Largemouth bass (*Micropterus salmoides*)

**DOI:** 10.1038/s41598-023-45645-8

**Published:** 2023-10-27

**Authors:** Jinxing Du, Jiaqi Shao, Shengjie Li, Tao Zhu, Hongmei Song, Caixia Lei, Meng Zhang, Yingkun Cen

**Affiliations:** 1 Key Laboratory of Tropical and Subtropical Fishery Resource Application and Cultivation, Pearl River Fisheries Research Institute, China Ministry of Agriculture, Chinese Academy of Fisheries Sciences, Guangzhou, 510380 China; 2 College of Fisheries, Henan Normal University, Xinxiang, 453007 China; 3 Jiyurunda Fishery Technology Co., Ltd, Foshan, 528203 China

**Keywords:** Protein sequencing, RNA sequencing, Animal breeding, Gene regulation

## Abstract

Acceptance of artificial pelleted diets contributes to increasing the cultured areas and output of carnivorous fish. However, the mechanism of acceptance of artificial pelleted diets remains largely unknown. In this study, the easy acceptance of artificial pelleted diets (EAD) group and the not easy acceptance of artificial pelleted diets (NAD) group of Largemouth bass (*Micropterus salmoides*) were divided based on the ratios of stomach weight/body weight (SB) after 0.5 h feeding, which was bigger than 18% in the EAD group and ranged from 8 to 12% in the NAD group. Through transcriptome and proteome sequencing, a total of 2463 differentially expressed genes (DEGs) and 230 differentially expressed proteins (DEPs) were identified, respectively. Integrated analyses of transcriptome and proteome data revealed that 152 DEPs were matched with the corresponding DEGs (named co-DEGs-DEPs), and 54 co-DEGs-DEPs were enriched in 16 KEGG pathways, including the metabolic pathways, steroid biosynthesis, fatty acid biosynthesis, etc. Furthermore, 3 terpenoid backbone biosynthesis-related genes (*Hmgcr*, *Hmgcs*, and *Fdps*) in metabolic pathways, 10 steroid biosynthesis-related genes (*Fdft1*, *Sqle*, *Lss*, *Cyp51a1*, *Tm7sf2*, *Nsdhl*, *Hsd17b7*, *Dhcr24*, *Sc5d*, and *Dhcr7*), and 3 fatty acid biosynthesis-related genes (*Acaca*, *Fasn*, and *Ascl*) were all up-regulated in the EAD group, suggesting that the lipid metabolism pathway and steroid biosynthesis pathway play important roles in early food habit domestication in Largemouth bass. In addition, the detection results of randomly selected 15 DEGs and 15 DEPs indicated that both transcriptome and proteome results in the study were reliable. Our study provides useful information for further research on the mechanisms of food habit domestication in fish.

## Introduction

Largemouth bass (*Micropterus salmoides*, LMB) is an important economic aquaculture fish in China and had an output of 702,093 tons in 2021, which is now believed to exceed one million tons in the next two decades^1^. As a carnivorous fish, LMB was traditionally cultured in the coastal areas and fed with fresh-frozen fish in China before 2019. This feeding system has some disadvantages, including insufficient seasonal supplementary, serious environmental pollution, and low conversion efficiency. Based on the growth trait and better receptiveness to artificial pelleted diet traits, a new variety of LMB “Youlu No. 3” was selectively bred in 2019. This variety can completely feed with artificial pelleted diets after domestication food of habit^2^, which promotes the extension of its farming areas from the coastal to inland areas. However, LMB fries perform different abilities to accept the artificial pelleted diets in the early food habit domestication process (domesticated from live food diets such as rotifer, zooplankton, and artemia to the artificial pelleted diets). Some LMB fries can quickly accept artificial pelleted diets, while some hardly adapt to artificial pelleted diets^2,3^. How to improve the food habit domestication efficiencies remains to be solved in LMB.

Similar to other traits such as growth and body color, food habit is also determined by genetic factors^4–6^. For example, in the domestication from carnivorous diets of wolves to starch-rich diets of dogs, mutations were observed in the genes involved in starch digestion and fat metabolism, including maltase-glucoamylase (*Mgam*), sodium/glucose cotransporter 1 (*Sglt1*), and alpha-amylase 2B (*Amy2b*)^5^. In Giant Panda (*Ailuropoda melanoleuca*), the pseudogenization of the taste receptor gene *T1r1* coincided with its dietary switch from carnivore to herbivore^7^. Several candidate genes and pathways involved in food habit domestication were also reported in fish. The expression of *T1r1* was significantly decreased during the food habit transition from carnivore to herbivore in Grass carp (*Ctenopharyngodon idella*), which might be related to the higher DNA methylation levels of *Cpg1* and *Cpg3*^8^. In Mandarin fish (*Siniperca chuatsi*), it was reported that metabolism, especially lipid metabolism, might contribute to the domestication of artificial diets, including genes such as stearoyl-CoA and triacylglycerol lipase^9^. Similarly, several genes in the lipid metabolism-related genes such as lipoprotein lipase (*Lpl*) and acyl-CoA synthetase long-chain family member (*Acsl1*) were reported to be the candidate genes for LMB fed on artificial pelleted diets^10^. However, the molecular mechanism of easy acceptance of artificial pelleted diets during the early food habit domestication in LMB remains to be further explored.

The transcriptomic analysis contributes to discovering the differential expressed genes and pathways in certain biological processes and developmental stages^11,12^. However, genes can produce multiple proteins through alternative splicing, RNA editing, and posttranslational modifications. Proteomic studies provide insight into changes in gene expression, protein localization, interactions, post-translational modifications, and turnover that can be difficult or impossible to obtain using other methods^13^. Currently, integrated transcriptomic and proteomic analysis has been widely applied to explore the functional genes and pathways in certain biological processes and developmental stages, such as the identification of salt resistance-related pathways and genes in *Solidago canadensis* L.^14^, stress response-related pathways and genes in Pearl oyster (*Pinctada fucata martensii*)^15^ and Chinese rare minnow (*Gobiocypris rarus*)^16^. In this study, to better explore the functional pathways and genes responsible for the easy acceptance of artificial pelleted diets of LMB, we conducted integrative analyses of transcriptome and proteome between the extreme populations of LMB fries during the food habit domestication process. Our study provided candidate pathways and genes responsible for the easy acceptance of artificial pelleted diets of LMB, which would benefit the improvement of food habit domestication efficiencies in the future.

## Results

### Transcriptome analysis between the EAD and NAD groups

**Figure 1:**
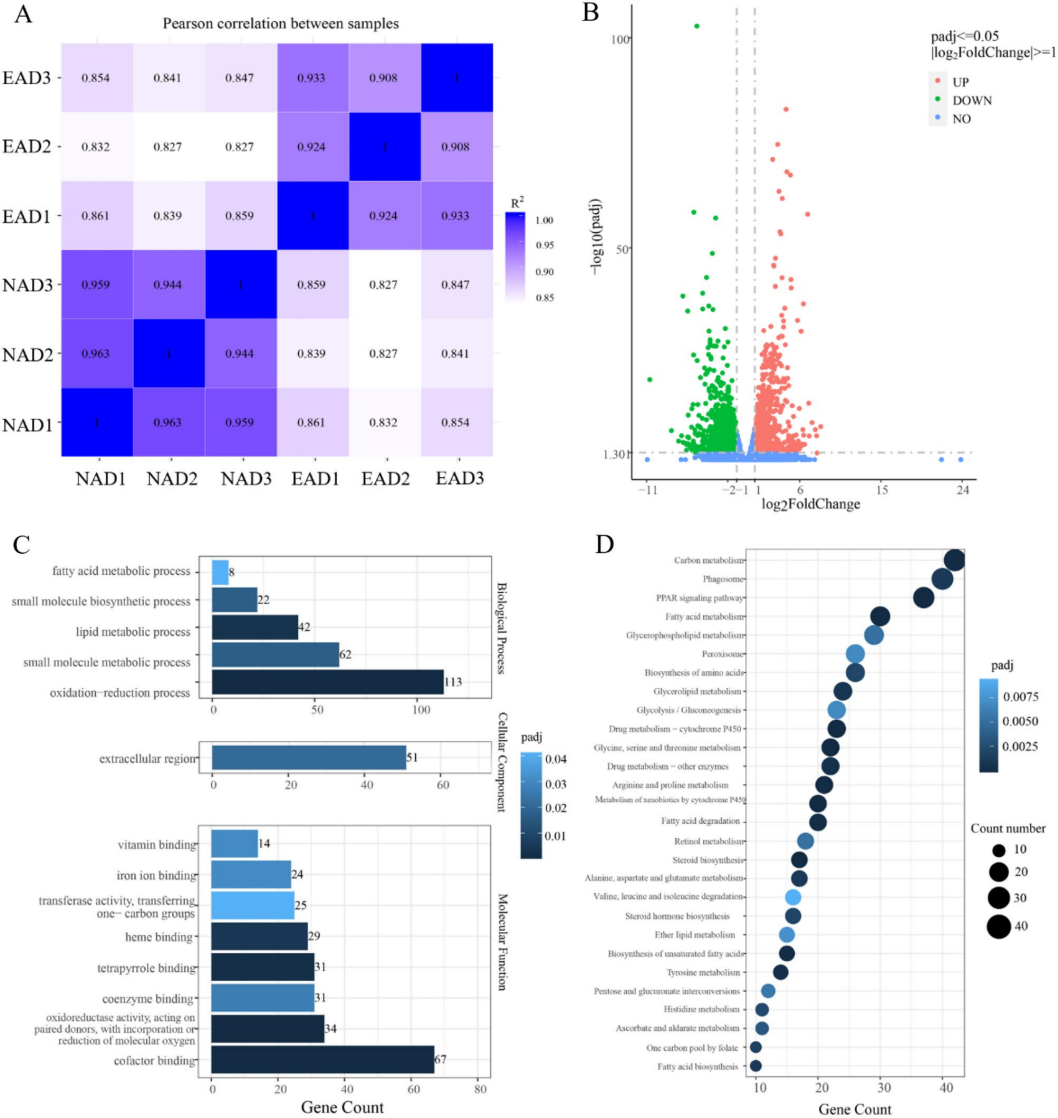
Person correlation analysis, volcano plot, GO terms analysis, and KEGG classification of differentially expressed genes between the EAD and NAD groups. (**A**) Pearson correlation analysis of the EAD and NAD groups. (**B**) The volcano plot of differentially expressed genes of the EAD and NAD groups. (**C**) GO terms analysis of differentially expressed genes. (**D**) KEGG classification of differentially expressed genes.

Six transcriptome libraries were constructed, and 127,176,480 and 131,442,004 raw reads were generated from the EAD group and the NAD group, respectively. After removing low-quality reads and adaptor sequences, 124,853,860 and 128,036,204 clean raw reads were obtained with 97.36–97.76% Q20 bases and 48.57–49.04% GC content. In total, more than 93% of clean raw reads were mapped and annotated to 25,210 genes of the LMB reference genome, and 84.17–88.14% of them were mapped to the unique locations in the reference genome (Table S1). Besides, the Pearson correlation analysis indicated that the unigenes’ expression patterns of the EAD group and the NAD group separated clearly (Fig. 1A).

Compared to the NAD group, 1222 up-regulated and 1241 down-regulated DEGs were identified in the EAD group, respectively (Fig. 1B, Tables S2, S3). These DEGs were assigned to 853 GO terms, including 50.53% biological process terms, 36.69% cellular component terms, and 12.78% molecular function terms (Table S4). Fourteen most abundant GO terms were assigned with a *P*-value less than 0.05, including 5 biological process terms, 1 cellular component term, and 8 molecular function terms (Fig. 1C). The top 3 GO terms in biological processes were oxidation–reduction process, small molecule biosynthetic process, and lipid metabolic process. The extracellular region was the most abundant cellular component. Cofactor binding, oxidoreductase activity, and coenzyme binding were the top 3 abundant molecular function terms (Fig. 1C). KEGG pathway enrichment analysis showed that these DEGs were enriched to 147 KEGG pathways (Table S5). After screening with a *P*-value less than 0.001, 43 significant enrichment pathways were identified, including carbon metabolism, phagosome, PPAR signaling pathway, steroid biosynthesis, fatty acid metabolism, glycerophospholipid metabolism, etc. (Fig. 1D).

### Proteome analysis between the EAD and NAD groups

**Figure 2:**
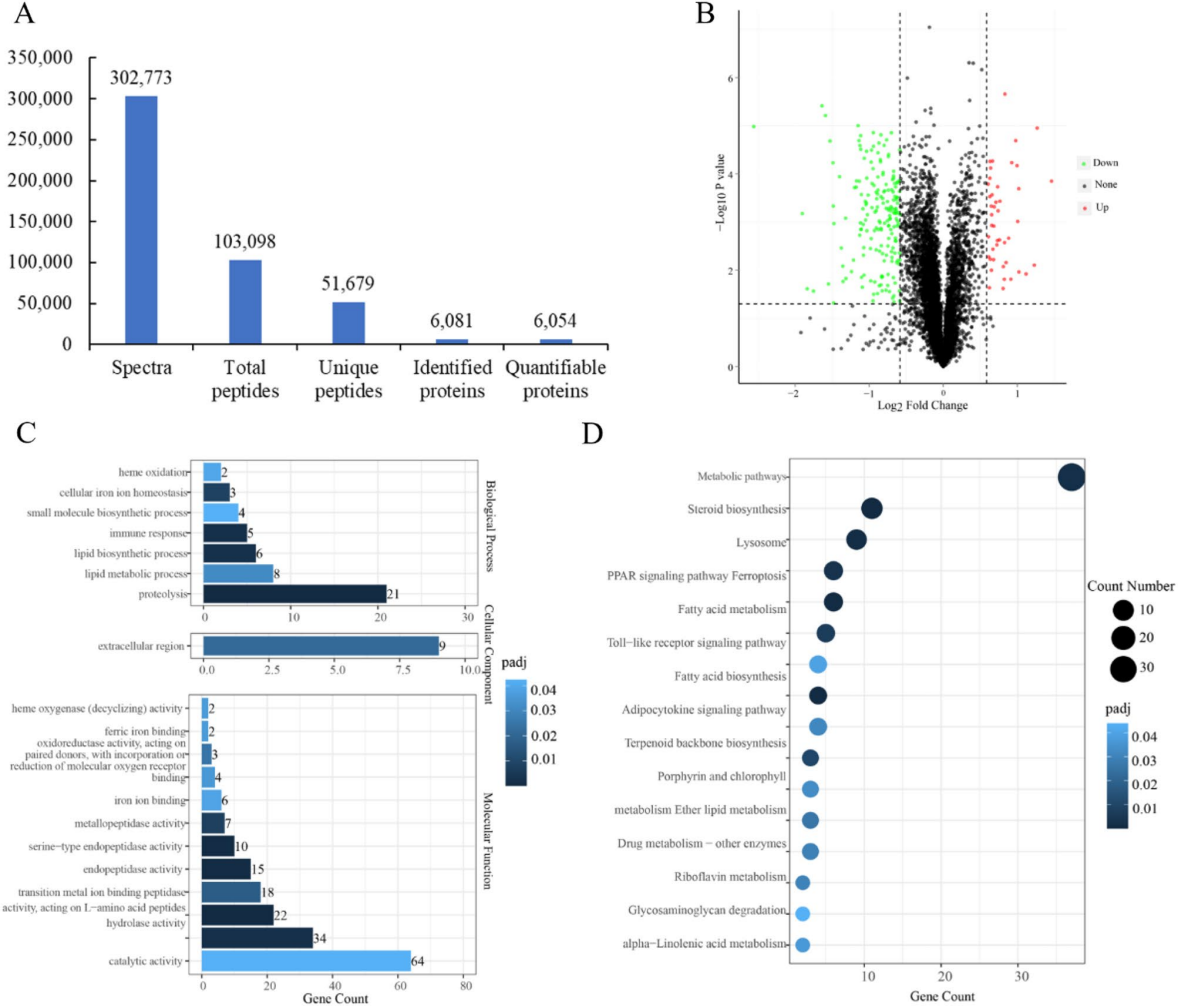
Statistics of mass spectrum data, volcano plot, GO terms analysis, and KEGG classification of differentially expressed proteins between the EAD and NAD groups. (**A**) Statistics of mass spectrum data. (**B**) The volcano plot of differentially expressed proteins of the EAD and NAD groups. (**C**) GO terms analysis of differentially expressed proteins. (**D**) KEGG classification of differentially expressed proteins.

In total, 302,773 spectra, which corresponded to 103,098 total peptides, 51,679 unique peptides, 6,081 identified proteins, and 6054 quantifiable proteins were determined via proteomic analysis (Fig. 2A). In comparison with the NAD group, 230 proteins had significant differential expression under *P* < 0.05 and fold change > 1.5 or < 0.67 in the EAD group, including 44 up-regulated DEPs and 186 down-regulated DEPs (Fig. 2B, Table S6). These 230 DEPs assigned to 61 GO terms, including 24.60% biological process terms, 6.55% cellular component terms, and 68.85% molecular function terms (Table S7). The top 3 GO terms in the biological processes were proteolysis, lipid metabolic process, and lipid biosynthetic process. The extracellular region was the most abundant cellular component. Catalytic activity, hydrolase activity, and peptidase activity, acting on l-amino acid peptides were the top 3 abundant molecular function terms (Fig. 2C). KEGG pathway enrichment analysis showed that these DEPS were annotated to 64 KEGG pathways (Table S8). After screening with a *P*-value less than 0.05, 15 significant enrichment pathways were identified, including the metabolic pathways, steroid biosynthesis, lysosome, PPAR signaling pathway, fatty acid metabolism, etc. (Fig. 2D).

### Correlations analysis between transcriptome and proteome data

**Figure 3:**
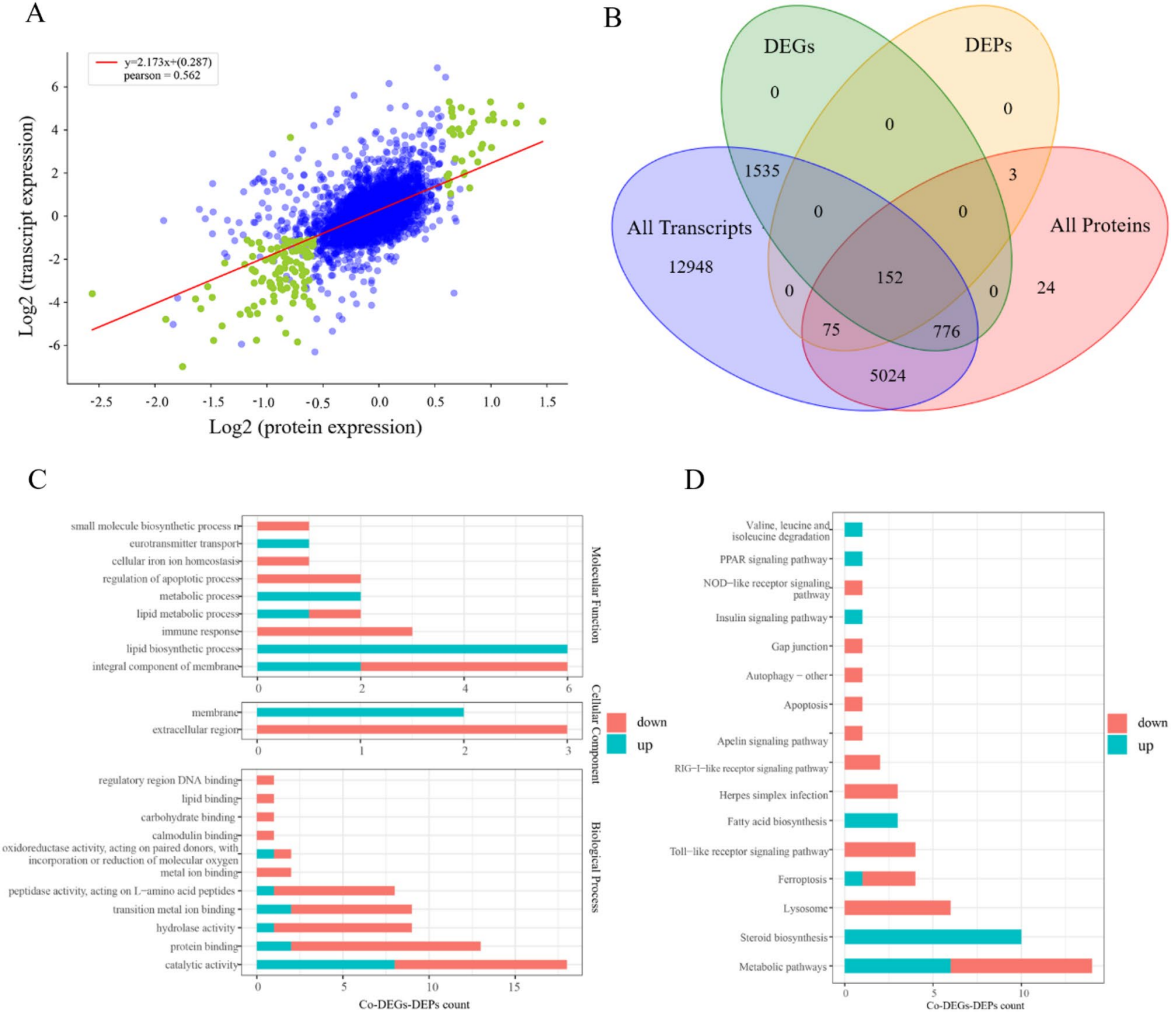
Pearson correlation coefficient analysis, GO terms analysis, and KEGG classification of co-DEGs-DEPs between the EAD and NAD groups. (**A**) Scatter plot of association analysis of transcriptomes and proteomes. The green dots represent proteins expressing significant differences and the blue dots expressing no significant differences. (**B**) Venn diagram representation of the number of co-DEGs-DEPs between the EAD and NAD groups. (**C**) GO terms analysis of co-DEGs-DEPs. (**D**) KEGG classification of co-DEGs-DEPs.

Pearson correlation coefficient of the transcriptomic and proteomic data was 0.562, suggesting some genes were significantly expressed at the mRNA level but not at the protein level (Fig. 3A). In comparison with the NAD group, a total of 152 corresponding DEGs and DEPs (co-DEGs-DEPs) were identified in the EAD group, including 39 up-regulated co-DEGs-DEPs and 113 down-regulated co-DEGs-DEPs (Fig. 3B, Table S9). GO enrichment analysis indicated that 94 of these 152 co-DEGs-DEPs were assigned to 22 GO terms (Table S9). The top 3 GO terms in the biological processes were integral component of membrane, lipid biosynthetic process, and immune response. The extracellular region was the most abundant cellular component. Catalytic activity, protein binding, and hydrolase activity were the top 3 abundant molecular function terms (Fig. 3C). KEGG pathway enrichment analysis showed that 54 of these 152 co-DEGs-DEPs were significantly enriched in 16 KEGG pathways (Table S9). The numbers of the genes involved in metabolic pathways and steroid biosynthesis were the 2 tops of all (Fig. 3D).

### Screening of candidate co-DEGs-DEPs responsible for the acceptance of artificial pelleted diets

**Figure 4:**
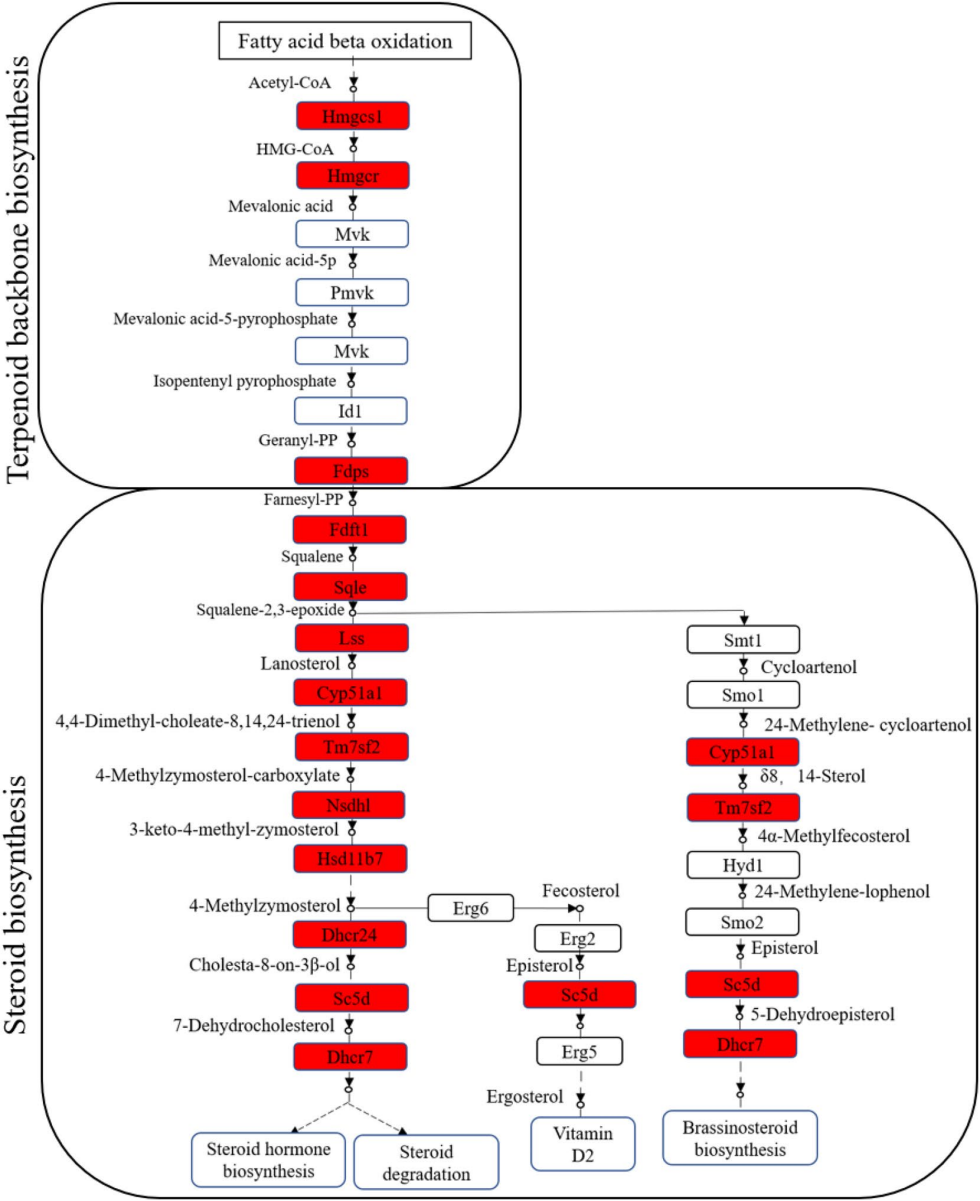
Terpenoid backbone biosynthesis and steroid biosynthesis pathway enriched in the liver of largemouth bass. The expression of terpenoid backbone biosynthesis-related genes 3-hydroxy-3-methylglutaryl-Coenzyme A reductase (*Hmgcr*), Hydroxymethylglutaryl-CoA synthase (*Hmgcs*) and Farnesyl diphosphate synthase (*Fdps*), and steroid biosynthesis-related genes Farnesyl-diphosphate farnesyltransferase 1 (*Fdft1*), Squalene epoxidase a (*Sqle*), lanosterol synthase (*lss*), Cytochrome P450, family 51 (*Cyp51a1*), Transmembrane 7 superfamily member 2 (*Tm7sf2*), NAD(P) dependent steroid dehydrogenase-like (*Nsdhl*), Hydroxysteroid (17-beta) dehydrogenase 7 (*Hsd17b7*), 24-dehydrocholesterol reductase (*Dhcr24*), Sterol-C5-desaturase (*Sc5d*), 7-dehydrocholesterol reductase (*Dhcr7*) were increased significantly in the EAD group. The red boxes represented the significantly up-regulated genes involved in the pathway.

According to the correlations analysis results, we mainly analyzed the co-DEGs-DEPs involved in the metabolic pathways and steroid biosynthesis. In the metabolism pathway, 6 up-regulated co-DEGs-DEPs and 8 down-regulated co-DEGs-DEPs were identified in the EAD group, respectively. The 6 up-regulated co-DEGs-DEPs included 3 terpenoid backbone biosynthesis-related genes: 3-hydroxy-3-methylglutaryl-Coenzyme A reductase (*Hmgcr*), Hydroxymethylglutaryl-CoA synthase (*Hmgcs*), Farnesyl diphosphate synthase (*Fdps*) (Fig. 4); 1 Taurine and hypotaurine metabolism-related gene Glutamate decarboxylase-like (*Gadl1*); 1 Pyruvate metabolism-related gene Acetyl-coenzyme A synthetase (*Acss2*), and 1 Ether lipid metabolism-related gene UDP glycosyltransferase 8 (*Ugt8*). The 8 down-regulated co-DEGs-DEPs included 3 Pyrimidine metabolism-related genes Thymidine kinase 2 (*Tk2*), Uridine phosphorylase 1 (*Upp1*), and Uridine phosphorylase 2 (*Upp2*); 2 urea cycle-related genes Arginase 1 (*Arg1*) and Arginase 2 (*Arg2*); 1 Glycolysis/Gluconeogenesis process-related gene Phosphoenolpyruvate carboxykinase (*Pck1*); 1 Ether lipid metabolism-related gene Phospholipase D family member 4 (*Pld4*); 1 Mucin type O-glycan biosynthesis-related gene Polypeptide N-acetylgalactosaminyltransferase 12 (*Galnt12*) (Table S9).

In the steroid biosynthesis pathway, there were 10 up-regulated co-DEGs-DEPs in the EAD group (*P* < 0.01, Table S7), including Farnesyl-diphosphate farnesyltransferase 1 (*Fdft1*), Squalene epoxidase a (*Sqle*), lanosterol synthase (*Lss*), Cytochrome P450, family 51 (*Cyp51a1*), Transmembrane 7 superfamily member 2 (*Tm7sf2*), NAD(P) dependent steroid dehydrogenase-like (*Nsdhl*), Hydroxysteroid (17-beta) dehydrogenase 7 (*Hsd17b7*), 24-dehydrocholesterol reductase (*Dhcr24*), Sterol-C5-desaturase (*Sc5d*), 7-dehydrocholesterol reductase (*Dhcr7*) (Fig. 4). Besides, there were 3 up-regulated co-DEGs-DEPs in fatty acid biosynthesis process, including acetyl-Coenzyme A carboxylase alpha (*Acaca*), fatty acid synthase (*Fasn*) and acyl-CoA synthetase long-chain family member (*Acsl*) (Table S9).

### Validation of the transcriptome and proteome data

**Figure 5:**
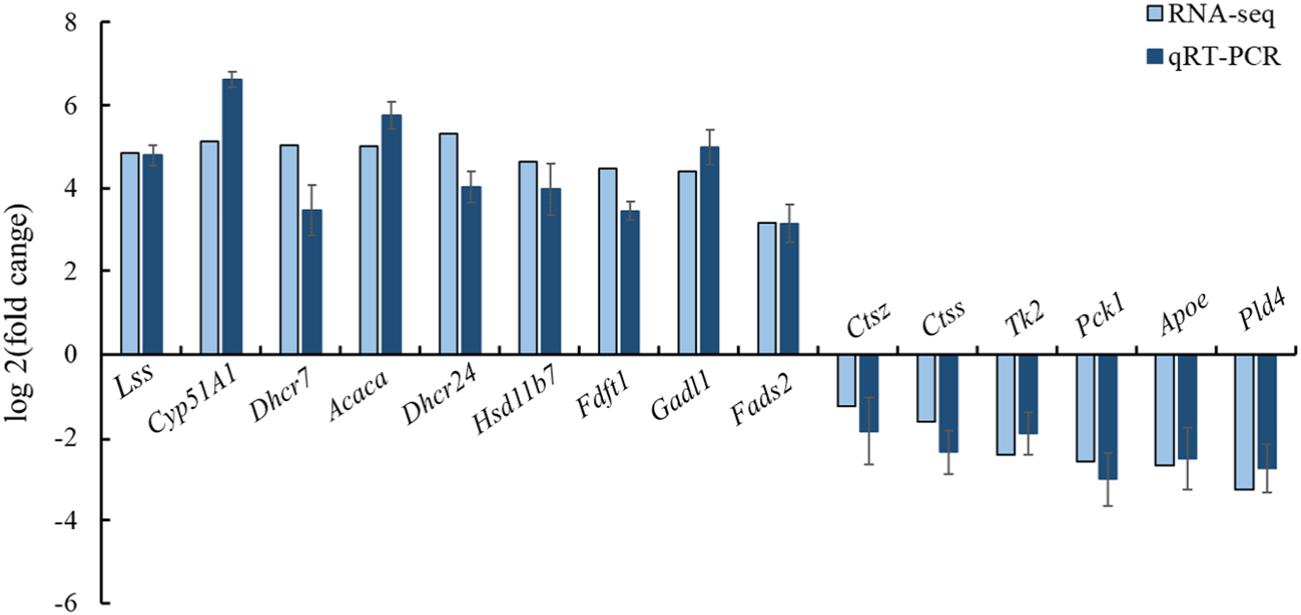
The qRT-PCR validation of 15 DEGs between the EAD and NAD groups.

In total, 15 candidate DEGs were selected and verified with the qRT-PCR method (Fig. 5). The results showed that the steroid biosynthesis pathway-related genes *Lss*,* Cyp51a1*, *Dhcr7, Dhcr24*, *Hsd17b7*, *Fdft1* were up-regulated in the EAD group. The fatty acid biosynthesis process-related gene *ACACA* was up-regulated in the EAD group. The metabolic pathways-related gene Glutamate decarboxylase-like (*Gadl1*) was up-regulated, whereas the *Pld4*, *Pck1*, and Thymidine kinase 2 (*Tk2*) were down-regulated in the EAD group. The PPAR signaling pathway-related gene fatty acid desaturase (*Fads2*) was up-regulated in the EAD group. The lysosome-related genes cathepsin (*Ctss*) and *Ctsz* were down-regulated in the EAD groups. Besides, the apolipoprotein E (*Apoe*), which is involved in the PPAR signaling pathway, was down-regulated in the EAD group. All these genes showed strong correlations with the corresponding RNA-seq data. Moreover, 15 corresponding DEPs were also selected for PRM analysis and which performed the same trends in abundance between the PRM and TMT quantification, including *Lss*,* Cyp51a1*, *Dhcr7, Dhcr24*, *Hsd17b7*, *Fdft1*, *Gadl1*, *Fads2*, *Ctss*, *Ctsz*, *Tk2*, *Pck1*, *Apoe* and *Pld4* (Table 1). In general, the trends in the expression changes measured by PRM and TMT were consistent.

**Table 1:** Comparison of PRM and TMT quantification results.

Protein description	Gene name	PRM	TMT
Lanosterol synthase	*Lss*	1.78	3.56
Cytochrome P450 family 51 subfamily A1	*Cyp51a1*	2.41	7.36
7-dehydrocholesterol reductase	*Dhcr7*	1.66	3.31
Acetyl-CoA carboxylase	*Acaca*	1.57	3.52
24-dehydrocholesterol reductas	*Dhcr24*	1.55	3.38
Hydroxysteroid 17-beta dehydrogenase 7	*Hsd17b7*	1.58	3.37
Farnesyl-diphosphate farnesyltransferase	*Fdft1*	1.97	5.94
Sulfinoalanine decarboxylase	*Gadl1*	2.75	11.41
Fatty acid desaturase 2	*Fads2*	1.88	3.78
Cathepsin Z	*Ctsz*	0.64	0.48
Cathepsin S	*Ctss*	0.63	0.46
Thymidine kinase	*Tk2*	0.54	0.17
Phosphoenolpyruvate carboxykinase 1	*Pck1*	0.59	0.24
Apolipoprotein E	*Apoe*	0.53	0.32
Phospholipase D4	*Pld4*	0.35	0.07

## Discussion

During the process of early food habit domestication in LMB fries, gradually increasing the proportion of artificial pelleted diets and prolonging the food habit domestication time are effective in improving the food habit domestication efficiencies. Currently, the average time of food habit domestication in LMB fries is about 7 to 10 days^2,3^. In this study, we distinguished the extreme populations according to the ratios of SB on the fourth day of food habit domestication, which was bigger than 18% in the EAD group and ranged from 8 to 12% in the NAD group, respectively. The results of Pearson correlation analysis of the transcriptome data indicated that this method was feasible. In comparison with the live food diets (such as zooplankton), it was reported that the artificial pelleted diets had more lipids and energy^9,17^. Moreover, artificial pelleted diets feeding regime to LMB is frequently associated with excess glycogen and lipid deposition, abnormal histology, inflammation, and apoptosis in the liver of fish^12,18^. The liver tissue, as one of the most important tissues in energy metabolism, is involved in the metabolism of carbohydrates, lipids, and proteins in animals^19^. Therefore, the liver tissues were selected as the targeted tissues in this study.

### Identification of candidate pathways responsible for the acceptance of artificial pelleted diets

Through transcriptome sequencing and proteomic analysis, 2463 DEGs and 230 DEPs were identified between the EAD group and the NAD group, respectively, and a total of 152 co-DEGs-DEPs were obtained through the correlations analysis of these DEGs and DEPs. KEGG pathway enrichment analysis indicated that 54 of the 152 co-DEGs-DEPs were significantly enriched in 16 KEGG pathways, in which the metabolic pathways (14 co-DEGs-DEPs) and steroid biosynthesis pathway (10 co-DEGs-DEPs) were the most enriched. In mandarin fish, it has been reported that metabolism, especially lipid metabolism, has been suggested to be responsible for the domestication of artificial diets^9^. Moreover, the steroid biosynthesis pathway is also a well-known component of lipid metabolism in animals^20^. Considering the lipids and energy differences between the live food diets and artificial pelleted diets^9,17^, the lipid and energy metabolism-related pathways were suggested to be the key factors responsible for the acceptance of artificial pelleted diets in LMB.

### Up-regulations of lipid metabolism-related genes promote the acceptance of artificial pelleted diets in LMB

We first analyzed the 14 enriched co-DEGs-DEPs in metabolic pathways, including 6 up-regulated co-DEGs-DEPs and 8 down-regulated co-DEGs-DEPs. Interestingly, 3 co-DEGs-DEPs involved in the terpenoid backbone biosynthesis process were up-regulated in the EAD group, which were *Hmgcs*, *Hmgcr*, and *Fdps*, respectively. The terpenoid backbone biosynthesis is one of the processes in metabolic pathways, which provides abundant precursors for complex secondary metabolite synthesis, including genes such as *Acat2*, *Hmgcs*, *Hmgcr*, *Mvk*, *Pmvp*, and *Fdps*^20,21^. *Acat2*, *Hmgcs*, *Hmgcr*, *Mvk*, and *Pmvp* are crucial enzymes in the mevalonate pathway, which is potentially the only pathway to produce isopentenyl-PP and dimethylallyl-PP precursors^20^. *Fdps* convert the precursors to farnesyl pyrophosphate and produce terpenoids^22^. Although there is no study about the function of terpenoid backbone biosynthesis in food habit domestication, it has been reported that terpenoid backbone biosynthesis plays a key role in the metabolism of animals under stressors. For example, exposure to dietary trivalent chromium up-regulated terpenoid backbone biosynthesis pathway-related genes *Hmgcr*, *Pmvk*,* Mvd*, *Id1*, and *Fdps* in Coral trout (*Plectropomus leopardus*)^23^, and low temperatures activated the genes including *Hmgcr*, *Hmgcs1*,* Acat2*, *Mvk*, and *Id1* in Grass carp (*Ctenopharyngodon idellus*)^24^. In this study, under the stimulation of different nutrients, these individuals who performed higher activity of terpenoid backbone biosynthesis could better metabolite the lipids in artificial pelleted diets. By contrast, the expression of the Glycolysis/Gluconeogenesis process-related gene *Pck1*, and urea cycle-related genes *Arg1* and *Arg2*^25^ were up-regulated in the NAD group. This phenomenon might be related to the metabolism of their body nutrients under starvation. In previous studies, it has been reported that short-term fasting treatment inhibited the Glycolysis-related genes Hexokinase (*Hk*) and pyruvate kinase (*Pk*) activity and enhanced the activity of gluconeogenesis-related genes *Pck1* in Nile tilapia (*Oreochromis niloticus*)^26^ and Black carp (*Mylopharyngodon piceus*)^27^; and long-term fasting treatment led the decreasing of fat and body protein in Gibel carp (*Carassius auratus gibelio*), which up-regulated the amino acids metabolism-related genes such as *Arg2*^28^. In this study, the LMB in the NAD group could not metabolize the lipids in artificial pelleted diets, and the starvation led to the decomposition of their stored protein and enhanced the gluconeogenesis in the liver to provide the energy, and caused the up-regulation of the *Pck1*, *Arg1*, and *Arg2* genes. Similarly, the Pyrimidine metabolism-related genes *Upp1*, *Upp2*, and *Tk2*, Mucin type *O*-glycan biosynthesis-related genes *Galnt12* and Ether lipid metabolism-related gene *Pld4* were also down-regulated in the NAD group, which could also be explained by the limitation of the lipid metabolism.

### Enhancement of the steroid biosynthesis pathway promotes the acceptance of artificial pelleted diets in LMB

Steroid hormones play critical roles in regulating nutrient metabolism in animals^29^. Currently, the steroid biosynthesis-related pathway has been reported in Coral trout^23^, Grass carp^24^, Friesian Cattle^30^, and Mice^31^, including genes such as *Fdft1*, *Sqle*, *Lss*, *Cyp51a1*, *Tm7sf2*, *Nsdhl*, *Hsd17b7*, *Dhcr7*, *Dhcr24*, *Sc5d*. *Fdft1* and *Sqle* are involved in regulating the conversion of mevalonate to cholesterol. Specifically, *Fdft1* (squalene synthase) catalyzes the conversion of two molecules of farnesyl pyrophosphate to squalene in a two-stage reaction^32,33^. *Sqle*, as one rate-limiting enzyme of the steroid biosynthesis pathway, catalyzes the squalene to 2,3-oxidosqualene (squalene epoxide), which is the first reaction of the cholesterol-specific biosynthetic pathway^34,35^. The *Lss* gene encodes the enzyme lanosterol synthase, which is one of the upstream precursors in the pathway of sterol biosynthesis for animals^36^. *Cyp51* gene specifically encodes for the lanosterol 14-alpha demethylase protein, which is a key enzyme for the conversion of lanosterol to cholesterol, and thus plays key roles in lipid metabolism and the steroid biosynthesis pathway^31^. *Hsd17b7* is an important enzyme required for cholesterol biosynthesis^37^. In previous studies, exposure to dietary trivalent chromium in Coral trout^23^ and low temperature treatments in Grass carp^24^ activated the steroid biosynthesis pathway, indicating the requirement for increased lipid metabolism in fish under stressors. In this study, under the stimulation of different nutrients, 10 steroid biosynthesis pathway-related genes were up-regulated in the EAD group, indicating these individuals could better convert the lipids in artificial pelleted diets to cholesterol through the enhancement of the steroid biosynthesis pathway.

### Enhancement of fatty acid biosynthesis pathway promotes the acceptance of artificial pelleted diets in LMB

Fatty acid biosynthesis is also one of the important processes in lipid metabolism, which converts the Malonyl-CoA de novo to fatty acids by several enzymatic activities^38^. *Fasn* is a multifunctional enzyme that catalyzes the de novo biosynthesis of fatty acids of 16 carbon atoms in length from acetyl-CoA and malonyl-CoA in the presence of NADPH, and alteration of this enzyme may affect economic traits including fatty acid composition^39,40^. Acetyl-CoA carboxylase is the flux-determining enzyme in the regulation of fatty acid synthesis in animal tissues. Currently, two isozymes of Acetyl-CoA carboxylase have been discovered in animals, acetyl-CoA carboxylase-a (*Acaca*) and b (*Acacb*)^41^. *Acaca* is involved in the first committed step of fatty acids synthesis and leads to the biosynthesis of long-chain fatty acids^42^, while *Acacb* controls the fatty acid β-oxidation by blocking fatty acid transport to the mitochondria^43^. Long-chain fatty acyl-CoA synthetases (Acsls) are a group of rate-limiting enzymes in fatty acid metabolism, which catalyzes the bioconversion of exogenous or de novo synthesized fatty acids to their corresponding fatty acyl-CoA by esterification^44^. In mandarin fish, it was suggested that the individuals who could accept artificial diets well might be attributed to the better capacity of glycerolipid metabolism and unsaturated fatty acids biosynthesis, including genes such as triacylglycerol lipase, stearoyl-CoA, and docosapentaenoic acid (DPA) metabolite^9^. In this study, 3 co-DEGs-DEPs (*Fasn*, *Acaca*, and *Acsl*) in the fatty acid biosynthesis pathway were up-regulated in the EAD group, indicating this group had a better ability to convert and store fatty acids in the artificial pelleted diets by the fatty acid biosynthesis pathway.

## Conclusion

In this study, through integrated transcriptome and proteome analyses, we provide powerful evidence that lipid metabolism and steroid metabolism are the most important processes responsible for the acceptance of artificial pelleted diets during food habit domestication in LMB. The metabolic pathways-related genes such as *Hmgcr*, *Hmgcs, Fdps*, steroid biosynthesis process-related genes such as *Fdft1*, *Sqle*, *Lss*, *Cyp51a1*, *Tm7sf2*, *Nsdhl*, *Hsd17b7*, *Dhcr7*, *Dhcr24*, *Sc5d*, and fatty acid biosynthesis process related genes such as *Acaca*, *Fasn*, *Acsl* were all up-regulated in the EAD groups, indicating that these individuals had better utilization effectiveness of the lipids in artificial pelleted diets. Therefore, further study will be conducted on the specific functions or mutations in these genes. Besides, according to these results, the addition of specific lipids in artificial pelleted diets might contribute to improving the food habit domestication efficiencies in LMB.

## Materials and methods

### Food habit domestication

A total of nearly 50,000 fries of “Youlu No. 3” LMB hatched on the same day were provided by Liang’s Aquatic Seed Industry Co., Ltd., Foshan, Guangdong, China. Since the sixth day after hatching, these fries were fed with Artemia (Artemia Aquaculture Co., Ltd, Tianjin, China) six times per day for 14 days. Then, these fries of similar size were randomly selected and divided into three tanks, with 5000 fries in each tank. During the food habit domestication process, the feeding amount of artemia (Artemia Aquaculture Co., Ltd, Tianjin, China) was gradually reduced and the powder food (50% crude protein, Fujian Tianma Science and Technology Group Co., Ltd, Xiamen, Fujian, China) was gradually increased. The period of the food habit domestication is about 7 days, and the fries were fed to satiation three times per day (8:00, 12:00, 17:00). The water temperature inside the cages was 26 ± 1 ℃ and the pH was about 7.5. The water was cleaned once per day.

### Sample collection

On the fourth day of food habit domestication, LMB fries were anesthetized with MS-222 (200 mg/L) after 0.5 h feeding, and their body weight and stomach weight (with the food intake) were measured. The extreme populations were distinguished according to the ratios of stomach weight/body weight (SB). The ratios of SB higher than 18% were suggested to be the group that easily accept artificial diets (EAD), and 8% to 12% SB were suggested to be the group that not easily accept artificial pelleted diets (NAD). Finally, 5 EAD individuals and 5 NAD individuals were identified in each replicate, respectively (Table S10). The livers were collected and stored at − 80 °C.

### RNA extraction, library construction, and sequencing

After equal-quality mixing, 3 EAD and 3 NAD mixed liver samples were obtained, respectively. Total RNA was extracted using the TRIzol Reagent (Invitrogen, Shanghai, China) according to the manufacturer’s instructions. The RNA integrity and quantity were determined using an Agilent 2100 Bioanalyzer (Agilent, Shanghai, China). The RNA-Seq libraries were constructed using the NEB Next® Ultra™ RNA Library Prep Kit for Illumina (NEB, USA). All six libraries were then sequenced on an Illumina NovaSeq 6000 sequencing platform and 150 bp paired-end reads were generated.

### Assembly, annotation, and analysis of differentially expressed genes

Raw reads were first quality-filtered using the Trimmomatic read trimming tool^45^. High-quality sequences were aligned and mapped to the reference genome of LMB (GenBank: GCA_019677235.1) using HISAT^46^. The gene expression levels were assessed by the Reads Per Kilobase per Million reads (RPKM) method^47^. Based on the obtained expression levels, significant differentially expressed genes (DEGs) between EAD and NAD groups were screened with criteria of false discovery rate (FDR) ≤ 0.05 and |log_2_fold change| > 1 by DEseq2^48^. The function and pathway of DEGs were analyzed by Gene Ontology (GO) and Kyoto Encyclopedia of Genes and Genomes (KEGG), respectively. In addition, the correlation among different samples used for transcriptome was analyzed by Pearson's correlation coefficient method^49^.

### Protein extraction

The samples used for proteome analysis were the same as those used for RNA sequencing. Total protein was extracted using the acetone extraction method^50,51^. Briefly, mixed liver samples were milled individually to a powder in liquid nitrogen and lysed with lysis buffer (100 mM NH_4_HCO_3_, 8 M Urea, pH 8). The homogenate was incubated with ultrasonication on ice for 5 min and centrifuged at 12,000×*g* for 15 min at 4 ℃. The supernatant was reduced in 10 nM DTT (DL-Dithiothreitol) for 1 h at 56 ℃ followed by adding sufficient iodoacetamide to the sample and incubating in darkness for 1 h at room temperature. Then the samples were completely mixed with 4 times the volume of precooled acetone and incubated at − 20 ℃ for at least 2 h. Samples were centrifuged at 12,000×*g* for 15 min at 4 ℃ and the precipitation was collected. After washing twice with 1 mL cold acetone, the pellet was dissolved by dissolution buffer (6 M Urea, 100 mM triethylammonium bicarbonate (TEAB), pH 8.5). The concentration and quality of the extracted protein were determined using the Bradford protein quantitative kit (Bio-Rad, CA, USA) according to the manufacturer’s instructions.

### Peptide preparation and TMT labeling of samples

120 μg of each protein solution was taken and the volume was made up to 100 μL with lysis buffer, 3 μL of 1 μg/μL trypsin, and 500 μL of 50 mM TEAB buffer added, the sample was mixed and digested at 37 °C overnight. An equal volume of 1% formic acid was mixed with the digested sample and centrifuged at 12,000×*g* for 5 min at room temperature. The supernatant was slowly loaded to the C18 desalting column, washed with 1 mL of washing solution (0.1% formic acid, 4% acetonitrile) 3 times, then eluted twice by 0.4 mL of elution buffer (0.1% formic acid, 75% acetonitrile). The eluents were dried by vacuum centrifugation. 100 μL of 0.1 M TEAB buffer was added to reconstitute, and 41 μL of acetonitrile-dissolved TMT (TMT® Mass Tagging Kits and Reagents, Thermo) labeling reagent was added, the sample was mixed with shaking for 2 H at room temperature. Then, the reaction was stopped by adding 8% ammonia. All labeling samples were mixed with equal volume, desalted, and lyophilized.

### Separation of fractions and LC–MS/MS analysis

TMT-labeled peptides were fractionated using a C18 column (Waters BEH C18 4.6 × 250 mm, 5 μm) on a Rigol L3000 HPLC at a flow rate of 1 mL/min. Gradient elution was developed by mobile phases A (2% acetonitrile, adjusted pH to 10.0 by using ammonium hydroxide) and B (98% acetonitrile, adjusted pH to 10.0 by using ammonium hydroxide). For transition library construction, shotgun proteomics analyses were performed using an EASY-nLC™ 1200 UHPLC system (Thermo Fisher, USA) coupled with a Q Exactive™ HF-X mass spectrometer (Thermo Fisher, USA). Full scan ranged from m/z 350 to 1500 with a resolution of 60,000 (at m/z 200), an automatic gain control (AGC) target value was 3 × 10^6^ and a maximum ion injection time was 20 ms. The top 40 precursors of the highest abundant in the full scan were selected and fragmented by higher energy collisional dissociation (HCD) and analyzed in MS/MS, where the resolution was 30,000 (at m/z 200) for 6 plex, the AGC target value was 50,000, the maximum ion injection time was 54 ms, a normalized collision energy was set as 32%, an intensity threshold was 120,000, and the dynamic exclusion parameter was 20 s.

### Proteome data analysis

The resulting spectra from each run were searched separately against the 809135-X101SC21062696-Z02-protein database (23,952 sequences) by the search engine Proteome Discoverer 2.4. The searched parameters are set as follows: mass tolerance for precursor ion was 10 ppm and mass tolerance for production was 0.02 Da. Carbamidomethyl was specified as fixed modifications, and Oxidation of methionine (M) and TMT plex were specified as dynamic modifications. Acetylation, TMT plex, Met-loss, and Met-loss + Acetyl were specified as N-terminal modification in PD 2.4. A maximum of 2 missed cleavage sites were allowed. To improve the quality of analysis results, the software PD 2.4 further filtered the retrieval results: Peptide Spectrum Matches (PSMs) with credibility of more than 99% were identified PSMs. The identified protein contains at least 1 unique peptide. The identified PSMs and protein were retained and performed with FDR no more than 1.0%. The protein quantitation results were statistically analyzed by T-test. Proteins with a *P* value less than 0.05 and fold change > 1.5 or < 0.67 were defined as differentially expressed proteins (DEPs)^52^. Besides, principal component analysis (PCA) was conducted between the EAD group and the NAD group to further verify the credibility of the proteome data.

### Functional analysis of proteins and DEPs

Gene Ontology and InterPro (IPR) functional analyses were conducted using the InterPro Scan program against the non-redundant protein database (including Pfam, PRINTS, ProDom, SMART, ProSite, PANTHER)^50^, and the databases of COG (Clusters of Orthologous Groups) and KEGG were used to analyze the protein family and pathway. DEPs were used for Volcanic map analysis and enrichment analysis of GO, IPR, and KEGG. The probable protein–protein interactions were predicted using the STRING-DB server^53^ (http://string.embl.de/).

### Correlation analysis between transcriptome and proteome data

To further confirm the correlation between transcript expression and protein expression, the Pearson correlations were calculated between the transcriptome and proteome. The DEGs and DEPs detected simultaneously in transcriptome and proteome based on the screening criteria respectively were referred to as co-DEGs-DEPs^52^. GO analysis and KEGG pathway enrichment analysis were performed to study the function of these co-expressed genes and proteins.

### Validation of transcriptome data by quantitative real-time PCR

The quantitative real-time PCR (qRT-PCR) method was applied to the validation of the reliability of the transcriptome sequencing data. The RNA samples used for the qRT-PCR validation were consistent with those used to construct the transcriptome library. The synthesis of cDNA was performed with a PrimeScript RT reagent Kit with gDNA Eraser (Takara, Japan) following the manufacturer’s instructions. The qRT-PCR was conducted by using SYBR Green Premix ExTaq (Takara, Dalian, China) in a CFX96 real-time PCR system (Bio-Rad, USA). The qRT-PCR was performed in a 20 μL reaction mixture including 10 μL SYBR Premix ExTaq™ II (2×), 0.4 μL of each primer (10 μM), 1 μL of cDNA, and 8.2 μL of ddH_2_O. The PCR procedure was as follows: 95 °C for 30 s, followed by 40 cycles of 95 °C for 5 s and 58 °C for 30 s, a 0.5 °C/5-s incremental increase from 58 to 95 °C, and 30 s elapse time for each cycle. The relative expression level was estimated using the 2^−ΔΔCt^ method^54^. A total of 15 DEGs were selected and validated, and *β-actin* was used as the internal reference gene to normalize the gene expression level (Table S11). Five biological and three technical replicates were used for each gene.

### Validation of proteome data by parallel reaction monitoring

To examine the reliability of protein expression levels obtained by TMT analysis, a total of 15 DEPs were selected and validated by parallel reaction monitoring (PRM) analysis. The proteins from the liver samples were extracted, tested, and digested with trypsin following the protocol for the TMT analysis. The obtained peptide mixtures were introduced into the mass spectrometer via a C18 Nano-trap column (2 cm × 75 μm, 3 μm) and then via a home-made analytical column (15 cm × 150 μm, 1.9 μm). The raw data obtained were then analyzed using Proteome Discoverer 2.2 (Thermo Fisher Scientific). The “missed cleavage” was set as 0, and 1–3 unique peptides were selected for each protein. Skyline 2.6 software was used for quantitative data processing and proteomic analysis.

### Ethics statement

The experiments involving largemouth bass in this study were approved by the Animal Research and Ethics Committee of the Institute of Hydrobiology, Chinese Academy of Sciences.

### Supplementary Information

Supplementary Table S1.

Supplementary Table S2.

Supplementary Table S3.

Supplementary Table S4.

Supplementary Table S5.

Supplementary Table S6.

Supplementary Table S7.

Supplementary Table S8.

Supplementary Table S9.

Supplementary Tables.

## Data Availability

The transcriptomic sequencing data have been deposited in the Sequence Read Archive (SRA) database with the accession number PRJNA1025875. The mass spectrometry proteomics data have been deposited to the ProteomeXchange Consortium (http://proteomecentral.proteomexchange.org) via the iProX partner repository with the dataset identifier PXD045975.
