# Supplementary material for: Integrated transcriptomic and proteomic analyses reveal the mechanism of easy acceptance of artificial pelleted diets during food habit domestication in Largemouth bass (Micropterus salmoides)
Source: Sci Rep. 2023 Oct 27;13:18461. doi: 10.1038/s41598-023-45645-8 (PMC10611700; doi:10.1038/s41598-023-45645-8)
Supplement: Supplementary file 1 — Supplementary Table S1. [file 41598_2023_45645_MOESM1_ESM.docx]

Table S1 Summary of the whole-genome resequencing data of the EAD group and the NAD group of Largemouth bass

| Group | Raw reads | clean reads | Q20（%） | GC content（%） | Map to genome | |
| --- | --- | --- | --- | --- | --- | --- |
|  |  |  |  |  | Total mapped ratio | Unique mapped ratio |
| NAD1 | 44013928 | 42968168 | 97.36 | 48.69 | 93.87% | 85.92% |
| NAD2 | 43530362 | 42552384 | 97.62 | 48.89 | 93.44% | 85.17% |
| NAD3 | 43897714 | 42515652 | 97.74 | 48.68 | 94.48% | 86.44% |
| EAD1 | 42294730 | 41440726 | 97.63 | 49.04 | 94.19% | 87.57% |
| EAD2 | 42714536 | 41928792 | 97.39 | 48.84 | 93.52% | 86.96% |
| EAD3 | 42167214 | 41484342 | 97.76 | 48.57 | 94.20% | 88.14% |
