# Supplementary material for: Integrated transcriptomic and proteomic analyses reveal the mechanism of easy acceptance of artificial pelleted diets during food habit domestication in Largemouth bass (Micropterus salmoides)
Source: Sci Rep. 2023 Oct 27;13:18461. doi: 10.1038/s41598-023-45645-8 (PMC10611700; doi:10.1038/s41598-023-45645-8)
Supplement: Supplementary file 10 — Supplementary Tables. [file 41598_2023_45645_MOESM10_ESM.docx]

Table S10 The ratios of stomach weight/ body weight (SB) of the EAD group and the NAD group

| **EAD group** | **Body weight /g** | **stomach weight /g** | **SB (%)** | **RAD group** | **Body weight /g** | **stomach weight /g** | **SB (%)** |
| --- | --- | --- | --- | --- | --- | --- | --- |
| 1 | 0.0694 | 0.0130 | 18.71 | 1 | 0.0474 | 0.0048 | 10.11 |
| 2 | 0.0775 | 0.0169 | 21.74 | 2 | 0.0466 | 0.0045 | 9.72 |
| 3 | 0.0684 | 0.0162 | 23.71 | 3 | 0.0514 | 0.0057 | 11.01 |
| 4 | 0.1074 | 0.0267 | 24.81 | 4 | 0.0382 | 0.0035 | 9.58 |
| 5 | 0.0731 | 0.0144 | 19.73 | 5 | 0.0309 | 0.0027 | 8.71 |
| 6 | 0.0669 | 0.0125 | 18.70 | 6 | 0.0501 | 0.0054 | 10.85 |
| 7 | 0.0851 | 0.0208 | 24.43 | 7 | 0.0449 | 0.0040 | 8.95 |
| 8 | 0.0662 | 0.0149 | 22.49 | 8 | 0.0367 | 0.0033 | 9.06 |
| 9 | 0.0949 | 0.0212 | 22.36 | 9 | 0.0317 | 0.0030 | 9.58 |
| 10 | 0.0860 | 0.0222 | 25.77 | 10 | 0.0379 | 0.0038 | 9.96 |
| 11 | 0.0858 | 0.0204 | 23.77 | 11 | 0.0384 | 0.0044 | 11.56 |
| 12 | 0.0915 | 0.0208 | 22.78 | 12 | 0.0410 | 0.0039 | 9.58 |
| 13 | 0.0829 | 0.0214 | 25.76 | 13 | 0.0308 | 0.0026 | 8.33 |
| 14 | 0.0680 | 0.0168 | 24.71 | 14 | 0.0410 | 0.0038 | 9.34 |
| 15 | 0.0981 | 0.0214 | 21.80 | 15 | 0.0396 | 0.0036 | 9.00 |

Table S11 Primers of 15 DEGs and internal reference gene of Largemouth bass

| Gene | Forward primer sequence (5′->3′) | Reverse primer sequence (5′->3′) |
| --- | --- | --- |
| *LSS* | GCCTCCGAGATGCCCATCAG | GGCCAATGGAGGGACAGAGC |
| *CYP51A1* | TGTTGCTGGCAGCCTCTGTC | AATGGCATGGCCCAGGAAGG |
| *HSD17B7* | GTTTGCGACGGCAGAGGGTA | CAGAGGCTCCAGCTCCCTGA |
| *FDFT1* | CTGCCACCGTATGGGAGTCG | CCTCCAGCTTGGACGCAGAG |
| *DHCR24* | GTGCGAGACATCCAGCGACA | GCCAGCGGCTCAACTCTCAA |
| *ACACA* | ACAGTCTCTGGGCACGACCT | CATCTGCACCAGCTGGCCTT |
| *DHCR7* | CTCGTACCGCTCCGCTGATG | GGGAGTATGTGGCCGAAGCC |
| *GADL1* | GGTCTGTGAGTGGCGTTCCC | GGCCGCTGTGGAATATGGCT |
| *FADS2* | ACATCACGCAGTGGGCCAAA | ATGGCTGGGCTCTGTTGCTG |
| *CTSS* | TGCCACACTCACTCCTCCCA | GAAGGCCCAGCAGGATCCAC |
| *APOE* | GAGCTCCAATCCCGCACCTC | CCAAGCCCTCAGCCTGTGTC |
| *PLD4* | AGCGCCAACATGGACTGGAG | CTGAGGCAGGGAGCTGTTGG |
| *CTSZ* | CGTGGCCGTCTGCCTATCTG | GGTGGTGCAGGTGCCACATT |
| *PCK1* | TGTGACGGCTCAGACGAGGA | GTCCTGGCCAACCAGCAGTT |
| *TK2* | GGCATCACGCTGCAGACCTA | AGCGTAGTCCACCTCTGGCA |
| *β-actin* | AAAGGGAAATCGTGCGTGAC | AAGGAAGGCTGGAAGAGGG |
